# Python tooth–inspired fixation device for enhanced rotator cuff repair

**DOI:** 10.1126/sciadv.adl5270

**Published:** 2024-06-28

**Authors:** Iden Kurtaliaj, Ethan D. Hoppe, Yuxuan Huang, David Ju, Jacob A. Sandler, Donghwan Yoon, Lester J. Smith, Silvio Torres Betancur, Linda Effiong, Thomas Gardner, Liana Tedesco, Sohil Desai, Victor Birman, William N. Levine, Guy M. Genin, Stavros Thomopoulos

**Affiliations:** ^1^ Department of Orthopaedic Surgery, Columbia University, New York, NY 10032, USA.; ^2^ Department of Biomedical Engineering, Columbia University, New York, NY 10027, USA.; ^3^ Department of Neurosurgery, Icahn School of Medicine at Mount Sinai, New York, NY 10029, USA.; ^4^ NSF Science and Technology Center for Engineering Mechanobiology, Washington University in St. Louis, St. Louis, MO 63130, USA.; ^5^ Department of Mechanical Engineering and Materials Science, Washington University in St. Louis, St. Louis, MO 63130, USA.; ^6^ Department of Biomedical Engineering, Washington University in St. Louis, St. Louis, MO 63130, USA.; ^7^ Department of Radiology and Imaging Sciences, Indiana University School of Medicine, Indianapolis, IN 46202, USA.; ^8^ Koru Medical Systems, Mahwah, NJ 07430, USA.; ^9^ Department of Mechanical and Aerospace Engineering, Missouri University of Science and Technology, St. Louis, MO 65409, USA.

## Abstract

Rotator cuff repair surgeries fail frequently, with 20 to 94% of the 600,000 repairs performed annually in the United States resulting in retearing of the rotator cuff. The most common cause of failure is sutures tearing through tendons at grasping points. To address this issue, we drew inspiration from the specialized teeth of snakes of the Pythonoidea superfamily, which grasp soft tissues without tearing. To apply this nondamaging gripping approach to the surgical repair of tendon, we developed and optimized a python tooth–inspired device as an adjunct to current rotator cuff suture repair and found that it nearly doubled repair strength. Integrated simulations, 3D printing, and ex vivo experiments revealed a relationship between tooth shape and grasping mechanics, enabling optimization of the clinically relevant device that substantially enhances rotator cuff repair by distributing stresses over the attachment footprint. This approach suggests an alternative to traditional suturing paradigms and may reduce the risk of tendon retearing after rotator cuff repair.

## Teaser

A device inspired by python teeth strengthened rotator cuff repairs and may reduce the risk of retearing in patients.

## INTRODUCTION

Rotator cuff tears are among the most prevalent tendon injuries, affecting more than 17 million individuals in the United States each year (*1*–*6*). The incidence of injury increases with age, as evidenced by more than 40% of the population over 65 years old experiencing a rotator cuff tear (*2*–*6*). These tears result in loss of shoulder strength, leading to pain, lost workdays, and limitations in recreational activities for patients (*5*, *7*–*9*). Rotator cuff tears typically occur at the tendon-to-bone insertion site, with the goal of rotator cuff repair being the anatomic restoration of the tendon attachment (*10*).

**Fig. 1.**
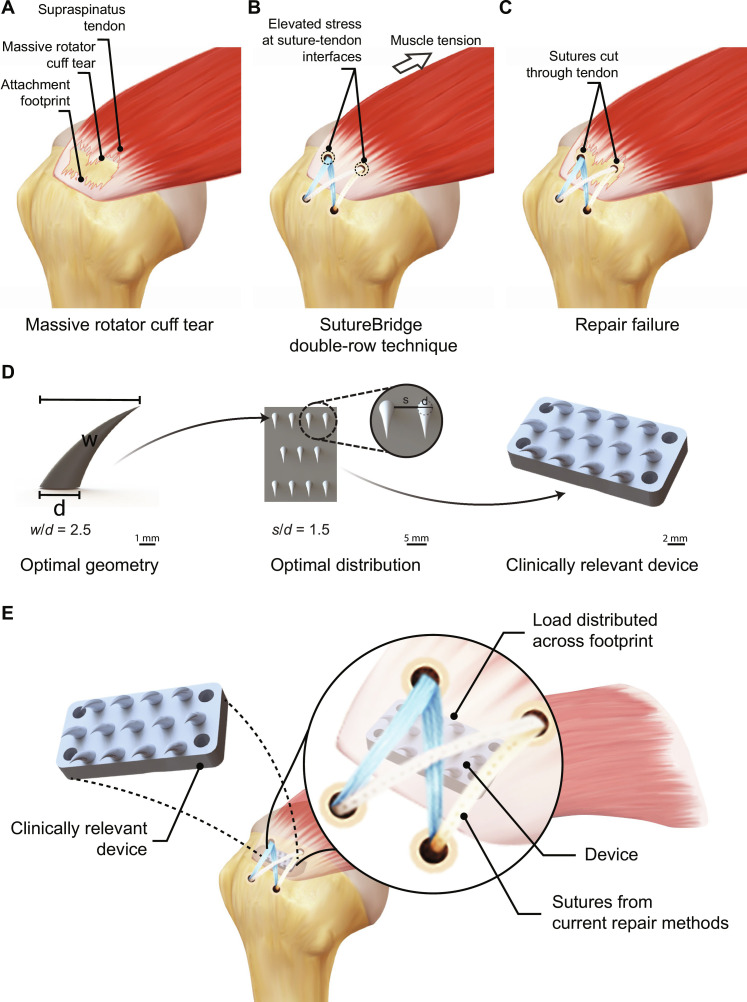
3D-printed biomimetic device for rotator cuff repair. (**A**) Schematic of a massive rotator cuff tear. (**B**) Schematic of a standard rotator cuff repair using sutures to repair tendon back to bone. (**C**) Schematic of repair failure due to sutures cutting through tendon. (**D**) Integrating simulations, 3D printing, and experiments, we determined the optimal grasping tooth shape and the optimal distribution of an array of grasping teeth and applied these results to develop a python tooth biomimetic device for rotator cuff repair. (**E**) The python tooth–inspired device interposed between tendon and bone significantly enhanced rotator cuff repair mechanics by improving stress distribution across the attachment footprint.

Rotator cuff surgical repair is the primary treatment for restoring shoulder function, with more than 600,000 procedures performed annually in the United States at a cost of $3 billion (*3*, *11*, *12*). However, successfully reattaching tendon to bone remains a significant clinical challenge. High failure rates occur following surgery, with rates increasing with patient age and tear severity. These rates range from 20% in younger patients with minor tears to a staggering 94% in elderly patients with massive tears (*13*–*15*). Rotator cuff repairs often fail due to sutures tearing through the tendon at the two or four grasping points where forces concentrate (Fig. 1, A to C).

Rotator cuff repair techniques have evolved over the past two decades, shifting from open surgery to arthroscopy and from manual knots to knotless suture anchor systems, reducing procedure time and costs (Fig. 1B) (*16*–*22*). Despite these advancements, the fundamental approach of sewing two tissues together has remained largely unchanged since at least ancient Egypt, still relying on sutures transferring tension at high-stress insertion points (*23*). Following tendon-to-bone reattachment surgery, sutures can tear through tendon at these points of high stress, a phenomenon referred to as “suture pull-through” or “cheesewiring,” leading to repair site gapping or rupture (Fig. 1, B and C) (*24*–*31*). Although advancements have been made to improve rotator cuff repair mechanics, including the use of modified repair configurations (*25*, *30*), suture “tape” (*32*), and orthobiologics (*33*–*38*), these advancements have not succeeded in reducing the retear rates postrepair. Current methods of suture anchor repair have reached a limit: Increasing the number of strands and anchors does not lead to improved outcomes, as demonstrated by studies showing that single-row repairs perform comparably with double-row repairs (*25*, *30*). Marginal improvements have been achieved using modified repair configurations and suture tape, which seek to provide more compression and minimize suture pull-through (*32*). More recently, orthobiologics have been used to stimulate healing (*33*, *35*–*37*). However, these approaches do not affect initial mechanical fixation, and their long-term biological benefits remain uncertain (*33*, *35*–*37*). In more recent years, US Food and Drug Administration (FDA)–approved reinforcement materials such as grafts, patches, or meshes have been used to provide additional mechanical support to the repair, with only modest improvements (*39*–*43*). Thus, there is a critical need for innovative and effective strategies to enhance rotator cuff repair mechanics and improve postoperative outcomes.

To address this need, we designed a biomimetic device, drawing inspiration from the relationship between tooth shape and gripping function observed in various predators. Snakes of the Pythonoidea superfamily grasp prey using teeth that are hooked and project inward, so that efforts by prey to escape pulls the teeth further into tissue, without tearing tissue (*44*–*46*). In contrast, certain shark teeth are triangular and serve to cut prey (*47*–*49*). Integrated finite element analysis and ex vivo experiments revealed relationships between tooth shape, tooth organization, and gripping mechanics that could be used to design a clinically relevant, three-dimensional (3D) printed, fixation device. This python-inspired device consisted of an optimized array of teeth and a base matching the curvature of the humeral head attachment site, with a profile that maintains compatibility with standard surgical techniques. Biomechanical testing demonstrated that the device nearly doubles the mechanical strength of state-of-the-art rotator cuff repair.

## RESULTS

### Curved teeth grasp rather than tear

**Fig. 2.**
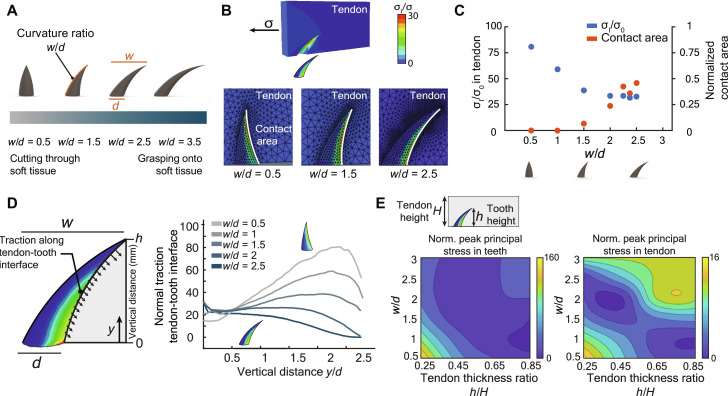
Optimization of single teeth. (**A**) Representative tooth geometries defined by the ratio *w*/*d*. (**B**) Finite element results of the interaction between teeth with various curvatures and tendon showing stress (color map) and contact area (white line). (**C**) Normalized maximum principal stress and normalized contact area for tooth geometries of *w*/*d* = 0.5 to 2.5 to disengage or tear through. (**D**) Traction along the tendon-tooth interface for tooth geometries of *w*/*d* = 0.5 to 2.5 (**E**) Peak stresses in tendon and teeth, as tendon thickness changes relative to tooth size. All parameters are dimensionless.

To test the hypothesis that tooth shape drives the balance between cutting and grasping of tendon, we studied tendon-tooth interactions using finite element analysis and ex vivo experimentation. Teeth were circular at the base (diameter *d*) and curved backward a distance *w* as they tapered to a point (Fig. 2A). Tooth designs studied spanned the range from shark-like to python-like by varying *w*/*d* (Fig. 2A). Simulations predicted that the peak principal Cauchy stress was highest in more conical teeth (low *w*/*d*) and decreased in teeth with greater curvature (Fig. 2, B and C). The contact area between the tooth and the substrate increased with increasing *w*/*d*, supporting the hypothesis that python-like teeth promote grasping (Fig. 2C). Contact area plateaued at *w/d* = 2.5, suggesting that a tooth with *w/d* = 2.5 would provide a balance between reducing stress and increasing contact area (to provide grasping) (Fig. 2C). Stresses along the tendon-tooth interface were more uniformly distributed for higher *w*/*d*, where stress concentrations were lower (Fig. 2D). Peak tooth stresses increased as tendon thickness increased relative to tooth size and decreased with increasing *w*/*d*, indicating that tooth size can be adjusted to the thickness of a specific tendon to optimize grasping strength (Fig. 2E).

**Fig. 3.**
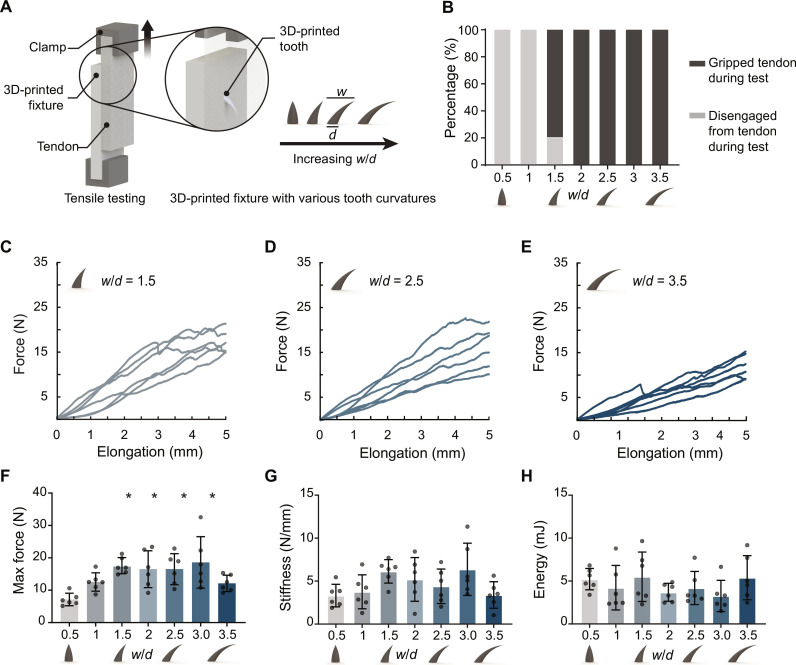
Mechanical characterization of single teeth with different curvature ratios. (**A**) Modified lap shear test setup for a single tooth. (**B**) Percentage of teeth engaging versus disengaging from tendon. (**C** to **E**) Force versus elongation curves for tooth geometries of *w*/*d* = 1.5, 2.5, and 3.5. (**F** to **H**) Peak force for up to 5 mm of tendon elongation, stiffness, and energy (*P < 0.05, when compared to *w*/*d* = 0.5). *N* = 6 biologic replicates per group. Mean values are shown, and error bars represent ± SD. Data were analyzed by a one-way analysis of variance (ANOVA) followed by Tukey’s post hoc tests.

To verify these predictions, we performed modified lap shear tests using 3D-printed teeth with prescribed *w/d* values inserted into bovine tendons. Results (Fig. 3) were consistent with the key predictions of the finite element simulations. First, the force required for the tooth to tear through the tendon increased to a plateau at approximately *w/d* = 1.5 (Fig. 3F), consistent with the decrease in peak stress observed in the finite element simulations to a plateau at approximately *w/d* = 1.5 (Fig. 2C). This plateau extended to *w/d* = 2.5 in both simulation and experiment. The experiments continued further, with less consistent results for *w/d* = 3 and *w/d* = 3.5 (Fig. 3, C to H). Second, like the finite element simulations, experimental observations were consistent with the hypothesis that curved teeth grasp better. The likelihood of a tooth completely disengaging from the tendon decreased markedly with increasing *w/d* (Fig. 3B), with no disengagement observed for *w/d* > 2. Analogously, in the simulations, contact area between the tooth and substrate reached a plateau at *w/d* = 2.25. We therefore chose *w/d* = 2.5 for the teeth that were studied subsequently.

### The distribution of teeth affects repair strength

**Fig. 4.**
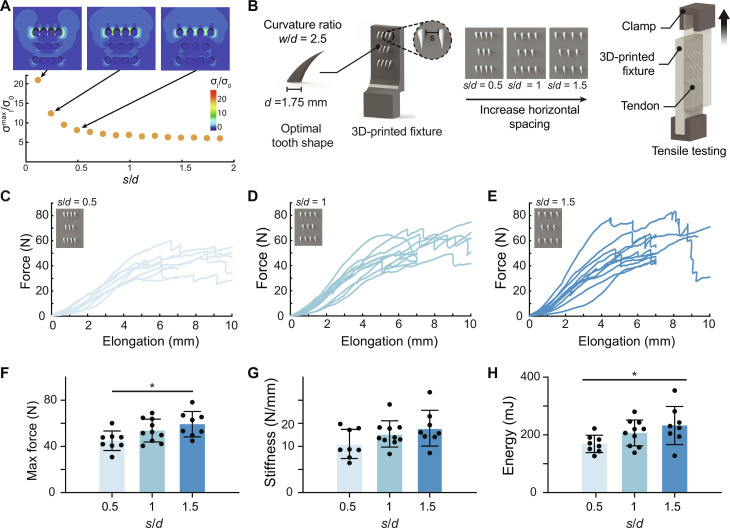
Mechanical characterization of tooth arrays. (**A**) Stress fields for three 2D tooth array patterns, with the following spacing between adjacent teeth: (i) *s*/*d* = 0.5, (ii) *s*/*d* = 1, and (iii) *s*/*d* = 1.5. Wider spacing led to a more uniform distribution of force among teeth, with the plateau beginning near *s*/*d* = 1.5. Parameters are dimensionless. (**B**) Schematic of lap shear tests for three array patterns. (**C** to **E**) Force versus elongation curves for patterns: *s*/*d* = {0.5, 1, 1.5}. (**F** to **H**) Effect of horizontal tooth spacing on maximum force, stiffness, and energy. Maximum force and energy increased with increasing tooth spacing, indicating that pattern *s* = 3 would have the highest tearing strength. Mean values are shown, and error bars represent ± SD. *N* = 8 to 10 biologic replicate per group. Data were analyzed by a one-way ANOVA followed by Tukey’s post hoc tests (*P < 0.05).

We hypothesized that tooth spacing of a clinically relevant tooth array would affect load distribution and thereby dictate the strength and energy absorption of the tooth-tendon attachment. Using the tooth shape defined in the previous section, three 3D tooth array patterns were studied numerically and in an ex vivo setting. Each tooth array was arranged in a consistent pattern. Teeth within the arrays were spaced uniformly, with the gap between them being *d*/2, *d*, or 3*d*/2, where *d* is the tooth diameter. Finite element analyses predicted that wider spacing resulted in more uniform distribution of force among teeth and increased the sharing of stresses across rows of teeth (Fig. 4A). This prediction was verified by experimental modified lap shear tests on arrays of teeth with *w*/*d* = 2.5 embedded in bovine tendon (Fig. 4B). In each of these tests, force-displacement curves began concave-up and then shifted to concave-down. The maximum force over 7 mm of displacement increased with tooth spacing [44.9 ± 8.5 N (*n* = 8), 53.8 ± 9.9 N (*n* = 10), and 59.3 ± 11 N (*n* = 8) for spacings *s*/*d* = 0.5, 1, and 1.5, respectively; Fig. 4, C to E]. Energy absorption also increased with *s* (Fig. 4, F to H). No significant differences in stiffness were observed between groups. While spacing the teeth further apart showed benefits, this adjustment was limited by the size of the attachment footprint at the repair site. Therefore, we did not further increase the spacing between the teeth.

### A biomimetic device doubles the surgical repair strength

**Fig. 5.**
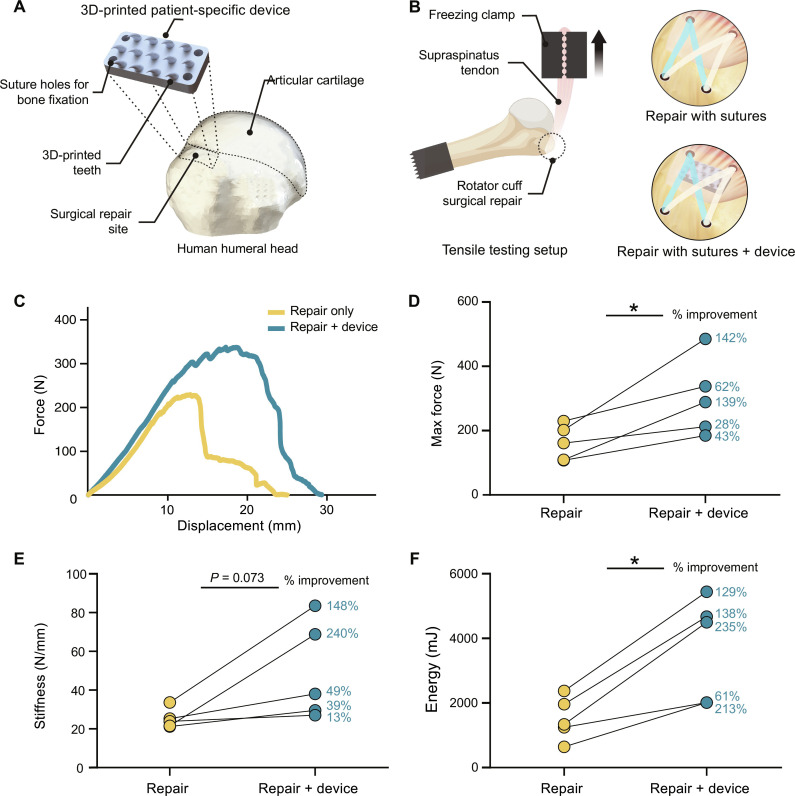
Biomimetic python tooth–inspired device nearly doubles the strength of rotator cuff repair. (**A**) 3D-printed python tooth–inspired device is placed at the native attachment site. (**B**) Schematic biomechanical testing setup for human cadaver rotator cuff tendons repaired with double-row suture only or device and suture. (**C**) Representative force-displacement curves for biomechanical tests of cadaver supraspinatus repairs with and without the device. (**D** to **F**) Mechanical evaluation of the device compared to the state-of-the-art rotator cuff repair. *N* = 5 biologic replicates per group. *P* values were determined using a two-tailed, paired Student’s *t* test (*P < 0.05). The device significantly increased the maximum force and energy absorption of the repaired rotator cuff.

Advancing toward translational application in clinical tendon-to-bone repair, we designed and 3D printed a biomimetic, rotator cuff-specific device using a biocompatible resin (Biomed Clear, Formlabs) (Fig. 5A). The biomimetic device consisted of an array of teeth atop a curved base. The base could be customized to match the patient-specific curvature of the humeral head at the supraspinatus tendon attachment site, as determined from computed x-ray tomography data (fig. S2). The device was designed to be secured to the humerus bone through four suture holes located at the corners of the rectangular base (fig. S7).

To evaluate the efficacy of the device compared to state-of-the-art rotator cuff repair, we conducted double-row suture anchor repairs on five paired cadaveric shoulders, with or without the device. For each of *n* = 5 shoulder cadavers, a rotator cuff tear was created at the supraspinatus tendon. Then, chosen blindly, one shoulder underwent standard double-row suture repair, while its paired cadaveric counterpart received the double-row suture repair along with the device (Fig. 5B). The repairs were loaded to failure using uniaxial tensile testing. Paired comparisons revealed that repairs incorporating the device exhibited an average increase in maximum force (i.e., strength) of 83% relative to matched controls without the device (Fig. 5D) and significantly greater energy absorption (fig. S9 and Fig. 5F). When adding the device, the failure mode shifted from the typical cheesewiring effect observed in standard repairs to midsubstance failure, where a portion of the tendon remained attached to the device. Postfailure inspections consistently showed that the device remained securely attached to the bone, with no breakage of any teeth.

## DISCUSSION

Our findings establish a python tooth–inspired approach for increasing repair strength immediately following rotator cuff repair surgery. The approach specifically addresses the main cause of high failure rates following traditional rotator cuff repair, namely, sutures pulling through the tendon due to tension at the medial side of the repair (*24*, *40*). Our device is positioned strategically between the two suture anchors in the medial row to strengthen these tendon-suture interfaces, which are prone to mechanical failure. The curved teeth added significant mechanical strength to the repair, due to what our simulations predicted to be a more even distribution of loads across the attachment footprint; the teeth grasp the tendon without tearing through the tissue. This approach may be adapted by changing the size and distribution of teeth as well as the base design to serve as a platform for enhanced repair of other connective tissues to bone (e.g., anterior cruciate ligament reconstruction and Achilles tendon repair). For high force attachments such as that at the Achilles enthesis, additional care might be needed in the design of teeth, such as rounding the base to reduce stress concentrations. In addition, in situations in which teeth might be in danger of breaking, a range of mechanisms from nature might be considered. Notable among these is the gomphosis, the peg-and-socket mechanism that allows teeth to move relative to the jaw at high stress and thereby reduce the likelihood of tooth or jaw fracture (*50*). Such a mechanism is attractive and will require additional optimization and analysis to account for how loss of stiffness of a tooth will affect the sharing of loads among other teeth.

Nature presents other examples of grasping systems analogous to teeth (*51*), including the burrs of hitchhiker plants (*52*), the prickles of roses (*53*), and the spines of asparagus (*53*). Burrs of the hitchhiker plant *Harpagonella palmeri* may be designed to distribute forces evenly and have been explored as inspiration for new suturing strategies (*52*). Prickles on roses come in a tremendous diversity of shapes and sizes, with some prickles of certain cultivars having a significantly recurved shape analogous to python teeth (*54*, *55*). Larger prickles in, for example, *Rosa arvensisi* may function to stabilize the plant against neighboring vegetation and appear to have a recurve *w*/*d* much less than the 2.5 that was found to be optimal in this study (*53*). This may suggest that in their interactions with soft tissues, they evolved to puncture rather than grasp flesh. Leaf-derived spines such as those of *Asparagus falcatus* and *Asparagus setaceus*, hook climbing plants that must grasp neighboring vegetation for mechanical stability after growing to a critical height, appear to have hooks that are far more recurved than those of pythons (*53*), perhaps suggesting that they serve exclusively to grasp.

Results add to prior studies of the functional role of tooth shape across species. Studies of shark tooth biomechanics demonstrate that specialized arrays of shark teeth, including tight ligamentous restraints against the jaw and optimized tooth orientation relative to the jaw, contribute to enhanced efficiency in tearing through tissue (*47*, *49*). Well-known relationships between tooth shape and specific diets demonstrate that tooth shape, including curvature, is adapted to accommodate feeding habits. Carnivorous species have complex and sharp occlusal surfaces for shearing meat, while insectivorous species feature simpler, blunt cusps for crushing insect exoskeletons (*56*). The current study extends this understanding by examining how tooth shape affects the balance between soft tissue tearing and grasping. Differences between triangular (shark-like) and curved (python-like) teeth suggest factors that enable posterior-curved teeth to facilitate the trapping of prey by pythons (*44*–*46*). Specifically, grasping is achieved through tooth curvature by reducing peak stresses and increasing contact between the jaw and the soft tissue. Grasping teeth must balance a trade-off between increasing contact area and mitigating stresses. Results showed that for insufficiently recurved teeth (that is, *w*/*d* < 2), a compressive force was required to ensure engagement; in the absence of compressive force restraining such a tooth against the tendon, the tooth could slide out of the tendon.

The number of sutures and their spacing is crucial to successful repair of the rotator cuff, where load transfer is typically concentrated at just two suture anchor points. Our studies suggest suture pull-through, a critical flaw of current repair techniques, can be addressed by increasing the number of attachment points, provided that all attachment points contribute to load bearing. This principle of load distribution is evident in tooth arrangements across species, with uniform tooth spacing believed to reduce risk of tooth fracture through efficient distribution of biting forces (*46*). Sharks continuously regenerate lost teeth, possibly in part for this purpose (*48*). Pythons have teeth that are less densely packed and, unlike many other species, have no differentiation between tooth types—all python teeth serve the same functional purpose (*46*, *57*, *58*). Our simulations suggest that this increased spacing may reduce peak tissue stresses and thereby protect against soft tissue rupture. In addition to these factors relating to gripping strength, sutures that are spaced too far apart may cause gapping across the repair site, whereas sutures positioned too close together risk inducing tissue necrosis (*23*).

The clinically relevant embodiment for rotator cuff tendon-to-bone repair was designed for compatibility with existing surgical methods. The device was positioned at the native supraspinatus tendon footprint between the greater tuberosity and the articular cartilage of the humerus, with a base manufactured to accommodate footprint sizes in patients (fig. S3) and teeth rising to grasp the tendon and distribute loads to lower stress concentrations and improve overall repair performance. The resultant doubling of repair strength could significantly affect postoperative outcomes by reducing the high rerupture rates now observed (*22*, *26*, *31*).

The success of rotator cuff repairs relies on both the mechanical strength provided by sutures and the application of biologics for tendon-to-bone healing. Despite the mechanical support offered by techniques such as the double-row suture bridge repair, the postsurgical failure rates remain alarmingly high. Biological approaches, including platelet-rich plasma, platelet-derived growth factors, and stem cells, show promise in promoting tendon-to-bone healing but lack mechanical reinforcement (*33*, *35*–*37*). More recently, FDA-approved devices, such as grafts, patches, or meshes, are being used to enhance the mechanical strength of rotator cuff repairs (*39*–*43*). However, these solutions have only shown modest mechanical improvements and do not target tendon-to-bone healing (*39*–*43*). Biomimetic tendon grasping represents a promising solution for rotator cuff repair, offering mechanical support and compatibility with standard of care, with the potential for localized drug delivery.

Our approach has several limitations. While the biomimetic device was 3D printed using a biocompatible resin, use of bioabsorbable materials may be preferable to improve long-term healing and reduce the risk of debris in the joint. In addition, the solid base of the device could be a barrier for tendon-bone integration. Future versions should consider a porous base that might better support tendon-to-bone healing and also serve as a depot for localized drug delivery. Our future studies will address these limitations and will refine this device using bioabsorbable materials and a porous structure to promote tendon-to-bone healing. We will also assess long-term outcomes through large animal model studies, investigating both mechanical integrity of the repair and healing. Overall, our research not only introduces a device that significantly improves mechanical strength, but also, in future design iterations, aims to facilitate the delivery of biologics using bioabsorbable materials with a porous structure to improve tendon-to-bone healing.

## MATERIALS AND METHODS

### Finite element analysis

#### Single tooth optimization

Idealized geometry: To quantify how teeth of different designs interact with tendon, 3D models of an isotropic tooth interacting with an orthotropic tendon were studied using finite element analysis in the Abaqus environment (Dassault Systèmes, Vélizy-Villacoublay, France). Teeth had circular cross sections and curved backward as they tapered to a point. The goal of the modeling effort was to determine first-order effects of how tooth shape could be varied to affect the stress distribution and contact area. Linear elasticity was adequate for capturing these first-order effects because the large strains that would necessitate hyperelasticity were evident only around the tips of teeth, in a small region where the nature of the appropriate hyperelastic constitutive law is unclear. Although strains could be high in the vicinity of the tips of teeth, the experimental validations supported that the linear modeling approach was effective for achieving the aims of optimizing the device performance. Seven tooth geometries were examined, with *w*/*d* = {0.5,1.0,1.5,2.0,2.5,3.0,3.5} (Fig. 2A). The interaction between the tendon and both the elastic tooth and rigid foundation were traction free. Because of symmetry, a half space was modeled, with tendon and teeth cut along the center plane shown (Fig. 2A), and symmetric boundary conditions were applied. Two different tendon thicknesses *H* were used, with the ratio of the tendon thickness *H* to the tooth height *h* being *H*/*h* = {1.33,2.67}. For the typical range of human supraspinatus tendon thicknesses, varying from *H* = 2 to 4 mm (*59*), this corresponds to teeth of heights *h* = 1.5 to 3 mm. All teeth had the same tooth base width of 1.5 mm and height of 3 mm. The tendon slab had dimensions 2 mm by 14 mm by *t* mm, with the tooth base centered at 0.92 mm along the length (Fig. 2A). Material properties: The tendon was modeled as linear elastic and transversely isotropic with modulus *E*_1_ = 450 MPa along the length of the tendon slab and *E*_2_ = 100 MPa transverse to it, and Poisson’s ratio ν_12_ = 0.55, and *G*_12_ = *E*_2_/2; the tooth was modeled as isotropic with *E* = 10 GPa and Poisson’s ratio ν = 0.3 (*60*–*62*). The longitudinal to transverse elastic modulus *E*_1_/*E*_2_ ratio was selected on the basis of literature to accurately represent the supraspinatus tendon’s biomechanical properties at the attachment, where the device is placed (*60*). This ratio is substantially smaller for the supraspinatus near its attachment than it is for other ligaments and tendons in the body. Boundary conditions: Traction parallel to the long direction of the tendon slab was applied to the tendon, pulling the tendon onto the tooth horizontally. The net pulling force was 0.96 N. Discretization: A convergence study was performed by increasing the number of quadratic interpolation tetrahedral elements until the stored energy converged to within 1%. This required finite element models with approximately 18,000 elements. Stress and displacement fields were recorded for further analysis. Input files are available for download.

#### Tooth array optimization

Idealized geometry: To optimize the spatial distribution of teeth, plane stress finite element analyses were conducted using models of rigid “teeth” of diameter *d* in a homogeneous, orthotropic “tendon” of size 8.93 *d* by 8.93 *d* (Fig. 4A). The primary goal of these simulations was to understand how tooth spacing affects the relative distribution of forces among the teeth during tendon loading. A 2D plane stress model capturing a representative cross section of the array proved sufficient for gaining these insights about the in-plane mechanics, the idea being that the teeth were all relatively stiff compared to the tendon, and the force distribution could thus be expected to be dominated by their 2D spatial disposition rather than the details of their 3D geometry. Three tooth array patterns were examined, with spacing *s* between adjacent teeth being *s*/*d* = {0.1,1.0,1.5}. Arranged in staggered rows, teeth in the second row were equidistant between the teeth in the first and third row (Fig. 4A). The first and third rows had four teeth while the second row had three teeth, the maximum number that could practically fit beneath the supraspinatus tendon at a tendon-to-bone attachment. The spacing between rows was kept constant at *d.* Material Properties: Tendon was modeled as orthotropic, as above. Boundary conditions: Traction was applied as shown (Fig. 4A) to achieve a 150 N force; all other boundaries were traction free. The interfaces between the rigid teeth, immovable, and the tendon were frictionless, with separation of the tendon from the teeth permitted. Discretization: A convergence study was performed, with convergence achieved for a mesh of approximately 10,000 eight-node biquadratic plane stress quadrilateral with reduced integration (CPS8R) elements. A free meshing algorithm with an advancing front was used. The maximum principal stress and the forces on each tooth were recorded (Fig. 2A). All equations were solved in the Abaqus finite element analysis environment (Dassault Systèmes, Vélizy-Villacoublay, France). Input files are available for download.

### Biomechanical testing

#### Single tooth optimization

To test the grasping capacity of single teeth in tendon, a modified single lap shear test was developed (Fig. 3A). Bovine deep digital flexor tendons (age 14 to 30 months; Animal Technologies, Tyler, TX) were fresh-frozen in phosphate-buffered saline–soaked gauze and stored at −20°C. Before testing, tendons were thawed overnight at 4°C and then cut into 10 cm by 6 cm by 0.8 cm planks using scalpel blades. Fixtures containing the seven different tooth geometries were 3D printed (EDEN 260VS, Stratasys Ltd.) in a stiff (*E* = 2.5 GPa) polymer (VeroWhitePlus, Stratasys, Rehovot, Israel). Each 3D-printed tooth shape (*n* = 6 per group) was inserted into a precut tendon block so that the entire tooth was fully engaged within the tendon but did not penetrate through the other side (fig. S1). To avoid slippage, the upper extremity of the bovine tendons and the bottom of the 3D-printed fixtures were secured using custom-made grips (Fig. 3A). A uniaxial tension test was then performed at 0.05 mm/s for up to 10 mm of displacement (ElectroForce, TA Instruments, Newcastle, DE). From the force elongation curves, peak force for 5-mm elongation, stiffness, and energy to yield were determined. Tooth engagement with the tendon was determined by visual inspection and verified through video captured during testing (movies S1 and S2).

#### Tooth array optimization

Similar testing methods were used to test the grasping capacity of three different teeth array patterns in bovine deep digital flexor tendon (9.91 cm by 6.10 cm by 0.318 cm, *n* = 8 to 10). Each fixture contained an array of 3D-printed teeth and was inserted into the precut tendon block so that all teeth were fully engaged within the tendon and did not penetrate through the other side (fig. S1 and Fig. 4B). A tensile test was performed at 0.05 mm/s for up to 7 mm of displacement (ElectroForce, TA Instruments, Newcastle, DE). From the force-elongation curves, peak force for 7-mm elongation, stiffness, and energy to 7 mm were determined. Tooth engagement with the tendon was determined by visual inspection and verified through video captured during testing.

### Biomechanical characterization

Stiffness was determined from the force-elongation curve using random sample correlation. Data were first trimmed to remove data below 10% and above 95% of maximum load to identify the region of interest. Then, two points were selected at random, and a line was drawn between them for *n* = 1000 iterations. All data points within a threshold range of 0.5% of the robust fit stress at the 80th percentile were considered as within an acceptable range of the best fit line. Of the *n* iterations, the iteration with the most inliers was deemed the best fit. This approach represents a “robust” fit, which, compared to a least squared errors fit, minimizes the effect of outlier points on the best fit line. The best fit was confirmed by visual inspection for each force-elongation curve. Energy was calculated as the area under the load-deformation curve up to the yield point.

### Device design and cadaveric fitting

To translate the idealized model and lap shear test results for clinical tendon-to-bone repair, a rotator cuff-specific device was designed using SolidWorks (Dassault Systèmes, Waltham, MA, USA). The device consisted of the optimized array of teeth, each 3 mm in height (fig. S6), placed in a curved base that matched the curvature and dimensions of a human humeral head supraspinatus tendon attachment site (17 mm by 10 mm footprint area) (*31*, *63*). The humerus 3D model used in this study was created from a deidentified patient computed tomography (CT) scan (approved by the Columbia University Institutional Review Board), processed into a 3D model using Mimics Innovation Suite (version 21.0.0.406, Materialise, Leuven, Belgium). The humeral attachment footprint was used as a mold to shape the device’s base. Given the generally flat nature of the attachment footprint, a model from a single patient was adequate to finalize the design. To address anatomical differences across patients, we designed multiple device footprints (15.5 mm by 6 mm to 17.5 mm by 8 mm) with 0.5-mm incremental adjustments. During cadaver shoulder tests, the surgeon selected the most suitable device size, similar to the standard clinical practice of choosing device sizes to match the patient’s anatomy.

The humerus 3D model used in this study was created from deidentified CT scans of patient data. The humerus’s attachment footprint from was used as a mold to shape the device’s base. Given the generally flat nature of the attachment footprint, a model from a single patient was adequate to finalize the design. To address anatomical differences across patients, we designed multiple device footprints (15.5 mm by 6 mm to 17.5 mm by 8 mm) with 0.5-mm incremental adjustments.

To check for the best design fit at the repair site, the following criteria were considered: (i) The device surface should match the attachment site surface, (ii) the device should not encroach on the articular cartilage, and (iii) the base thickness should be no more than 2 mm (figs. S4 and S5). The fit of the device at the repair site and the grasping ability of the teeth were evaluated in human cadaver shoulders for five different device prototypes (figs. S4 and S5). These tests led to subsequent adjustments in the design of the device that informed the final device design implemented in cadaver tests. Human cadaver shoulders were obtained from Anatomy Gifts Registry (Anatomic Gift Foundation Inc., Hanover, MD).

### Human cadaver rotator cuff repairs

To assess the device in a clinically relevant rotator cuff repair setting, paired human cadaver rotator cuff samples were used (Anatomic Gift Foundation Inc., Hanover, MD). Clinically relevant supraspinatus tendon tears were created with a scalpel and then repaired using a double-row suture bridge technique, in a paired fashion either with or without the device (*n* = 5 per group). After repair, humerus-supraspinatus tendon-muscle samples were carefully isolated and stored at 4°C overnight (fig. S8).

### Biomechanical testing of cadaveric specimens

The humerus was secured in a pipe with two orthogonal k-wires and Rockite cement (Hartline Products Co. Inc., Cleveland, OH). The pipe was secured close to the humeral head to prevent flexion. The rotator cuff muscle was secured in a freezing clamp using liquid CO_2_. The humerus was angled at 120° relative to the tendon so that muscle was pulled parallel to the tendon fibers at the insertion. Repaired supraspinatus samples were held in tension at 15 N for 20 s and then pulled in uniaxial tension to failure at 0.5 mm/s (movies S3 and S4 and fig. S8). Force and grip displacement data were recorded (MTS Systems Corporation, Eden Prairie, MN, USA), and maximum force, stiffness, and energy to failure were determined.

### Statistical analysis

Details of the sample size and appropriate statistical test are included in the figure captions. All data are shown as mean ± SD. Statistical analysis for all experiments was performed in GraphPad Prism 7 software. The threshold for statistical significance was defined at *P* < 0.05.
