# Supplementary material for: Python tooth–inspired fixation device for enhanced rotator cuff repair
Source: Sci Adv. 2024 Jun 28;10(26):eadl5270. doi: 10.1126/sciadv.adl5270 (PMC11212709; doi:10.1126/sciadv.adl5270)
Supplement: Supplementary file 1 — Supplementary Text Figs. S1 to S9 Legends for movies S1 to S4 Legend for data file S1 [file sciadv.adl5270_sm.pdf]

Science Advances
AAAS

Supplementary Materials for

Python tooth–inspired fixation device for enhanced rotator cuff repair

Iden Kurtaliaj *et al.*

Corresponding author: Stavros Thomopoulos, sat2@columbia.edu; Guy M. Genin, genin@wustl.edu

Sci. Adv. **10**, eadl5270 (2024)
DOI: 10.1126/sciadv.adl5270

The PDF file includes:

Supplementary Text
Figs. S1 to S9
Legends for movies S1 to S4
Legend for data file S1

Other Supplementary Material for this manuscript includes the following:

Movies S1 to S4
Data file S1

Supplementary Text

Patient-specific implant design

The humeral head model used for baseline study was generated from an anonymized computed tomography (CT) scan of a single patient. CT data were obtained with Institutional Review Board approval at the Columbia University New York-Presbyterian Hospital and anonymized. CT images of the humerus were processed using the Mimics Research software package (v.21.0.0.406, Materialise NV, Leuven). Segmentation by thresholding, multiple-slice editing, and auto-interpolation was performed. A drawing exchange format (.dxf) 2D image of the patient's humerus was then imported into Autodesk Meshmixer (Autodesk, San Rafael, CA) and smoothed to reduce the file size and to create a 3D model that is compatible with Solidworks v2019 (Dassault Systèmes, Vélizy-Villacoublay). The 3D humerus geometry was used as a mold to design a clinically relevant device using Solidworks. The final prototype of the device consisted of a rectangular base with gripping teeth of a shape that was optimized as described in the article. The posterior side of the base conformed to the geometry of the humerus at the tendon attachment where the device was placed, making the device patient-specific. The device's final design was saved as an STL file and fabricated from a biocompatible resin (Biomed Clear, Formlabs, Somerville, MA) by 3D printing (Form 3, Formlabs). Given the relatively flat presentation of a typical attachment footprint, a model from a single patient was adequate to finalize the design. To accommodate anatomical differences between male and female glenohumeral joints, we incorporated modular implant sizing offering footprints ranging from 15.5 x 6 mm to 17.5 x 8 mm in 0.5 mm increments.

Device dimension constraints

Functional evaluation tests for fit at the attachment of five different design iterations of the device were performed in a cadaver biomechanics lab using shoulder cadavers. Before testing the devices, surgeons were instructed to create a massive rotator cuff injury in the shoulder similar to the pathology seen in patients and then insert the device in between the tendon and bone. The results of these tests informed subsequent design modifications and confirmed that the base of the device should be constrained within the dimensions of the attachment (0.67” x 0.4” (17.0 x 10.2 mm) footprint area (*31,63*) and not encroach on articular

cartilage, and that the height of the base needed to be constrained to less than 2 mm. Design within these dimensions does not limit the normal shoulder range of motion. To account for anatomic variations in glenohumeral joints of male vs. female, different device footprints were designed, ranging from 15.5 x 6 mm to 17.5 x 8 mm with 0.5 mm increments in width and length.

Parameterization of tooth shape

The geometry of teeth was defined by two parameters involving the ratio tooth curvature and length:height ratio. Tooth curvature was found to define the ability of teeth to grasp onto soft tissue without damaging it. Tooth length:height ratio affected the grasping ability of the teeth, with increased length increasing the contact area between the tooth and soft tissue up to a threshold. The height of the teeth was limited by the average thickness of the supraspinatus tendon, which varies from 4.9 - 5.6 mm in healthy patients (*59*). Thus, the largest tooth height permissible that can grasp the tendon without tearing through the other side was experimentally determined.

Two identical devices with different tooth heights (2 mm and 3 mm) were biomechanically tested in shoulder cadavers, and force-displacement curves were recorded. A qualitative assessment was made to ensure that the device did not puncture through the tendon. As seen in the data (Fig. S6), theoretical calculations were verified, with the taller teeth having the higher failure load.

Fixation onto bone

The standard for rotator cuff repair is the double-row suture-bridge repair technique, which involves re-attaching the torn or injured tendon to the humerus using suture anchors (16). The anchor component is typically bullet-shaped and has ridges that help tunnel the anchor into the bone. The suture threads are passed through the tendon and tied to the anchors. Secure fixation of the device into bone and compatibility with the current surgical procedure are crucial features for translating the device.

Different methods of securing the device into bone (e.g., an add-on design to current suture anchors, additional fixation screws, and additional sutures) were considered and functionally tested in cadavers. Predetermined distances between suture anchors in a row in the

case of an add-on design or introducing new suture anchors to secure the device into bone were not ideal for clinician use, thus making these design choices not compatible with the current standard of rotator cuff repair. The final prototype was fixed into the bone through 4 suture holes placed in the four corners of the rectangular base. In the medial row, additional sutures from suture anchors were passed through the device's medial holes, and knots were tied based on the surgeon's preference. In the lateral row, suture hook threads were used to pass the additional sutures from the lateral suture anchors through the lateral holes of the device. The lateral suture anchors containing the sutures from medial suture anchors and sutures from the lateral holes of the device are tunneled into the bone, thus finalizing the fixation of the device into the bone.

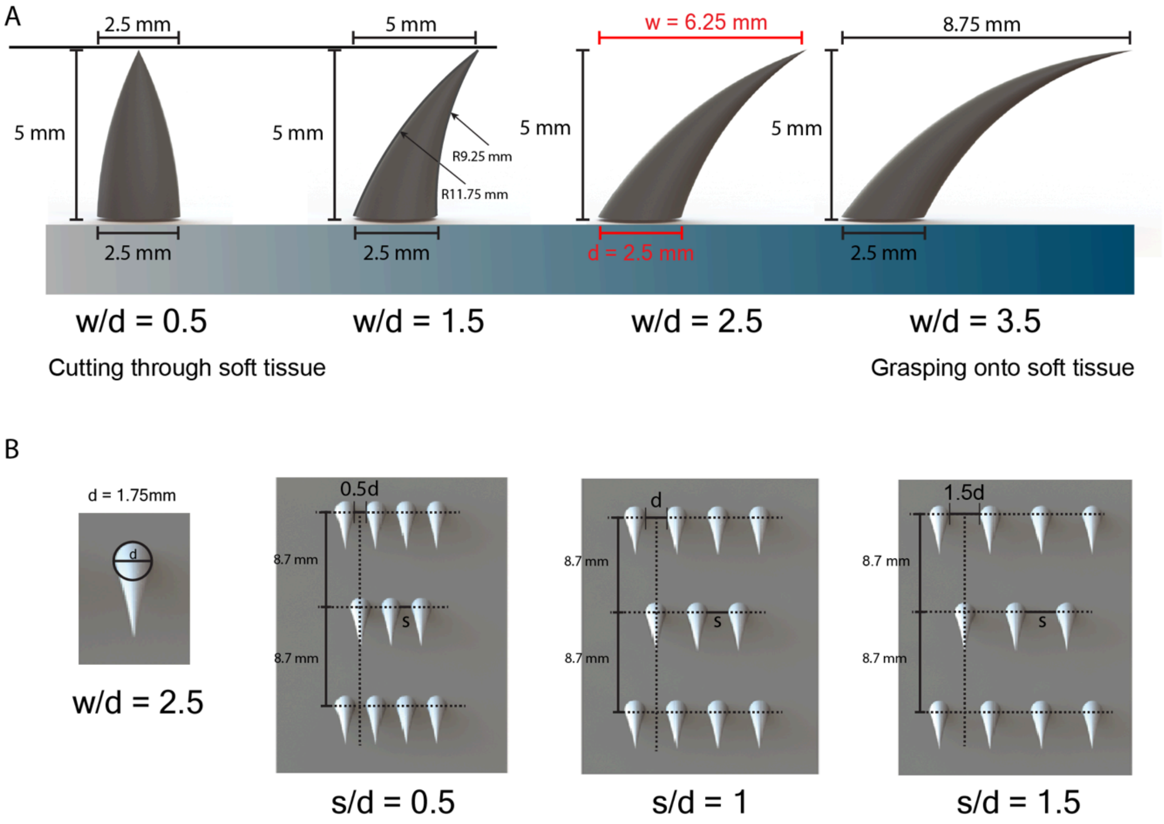

Fig. S1. Dimensions of 3D printed fixtures for modified lap shear tests.

(**A**) Dimensions of 3D-printed teeth with various curvature ratios used in modified lap shear tests. (**B**) Dimensions of 3D-printed teeth arrays with incremental spacing between teeth in each row used in modified lap shear tests

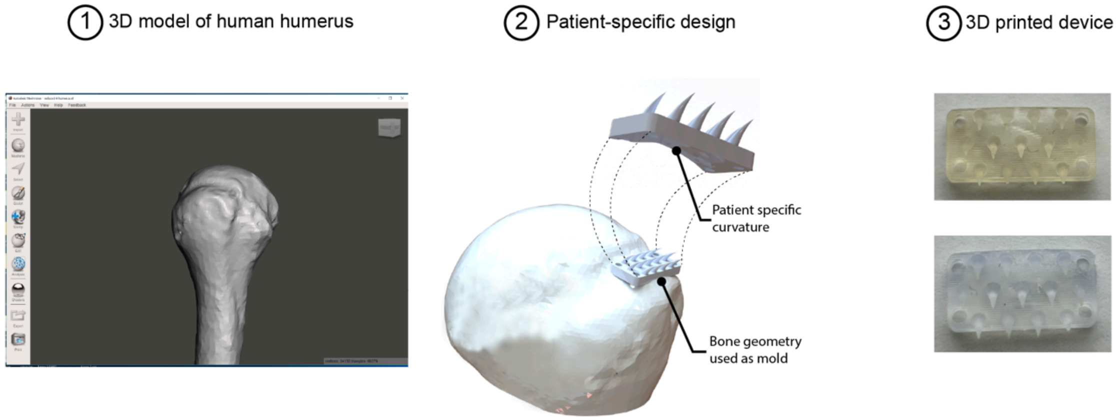

Fig. S2. The workflow chart for fabricating a patient-specific device for rotator cuff repair. A human humerus 3D model was used to contour the posterior side of the base so that the device could perfectly fit at the tendon-to-bone attachment.

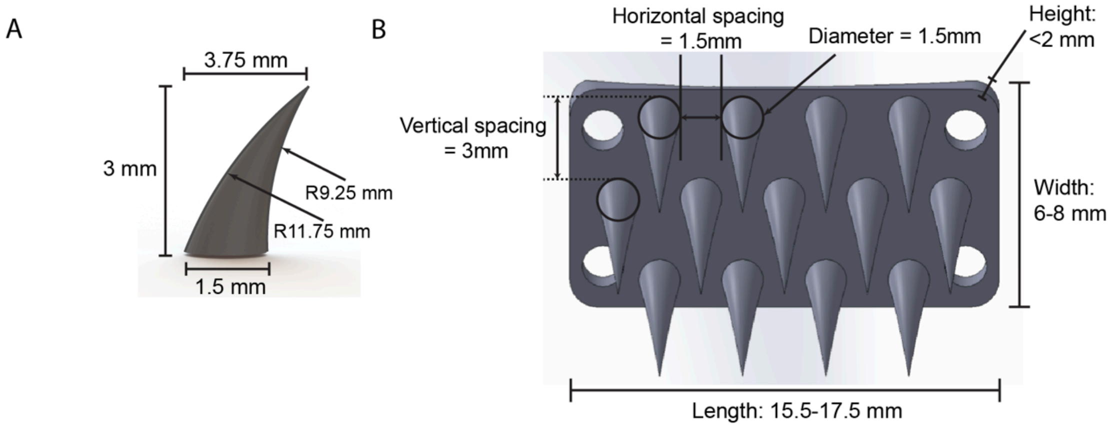

Fig. S3. Dimensions of the 3D-printed device for cadaver tests.

(**A**) Dimensions of 3D-printed teeth. (**B**) Dimensions of 3D-printed device used in cadaver tests. Different footprints ranging from 15.5 x 6 mm to 17.5 x 8 mm with 0.5 mm increments in width and length were used in cadaver tests based on the surgeon’s discretion.

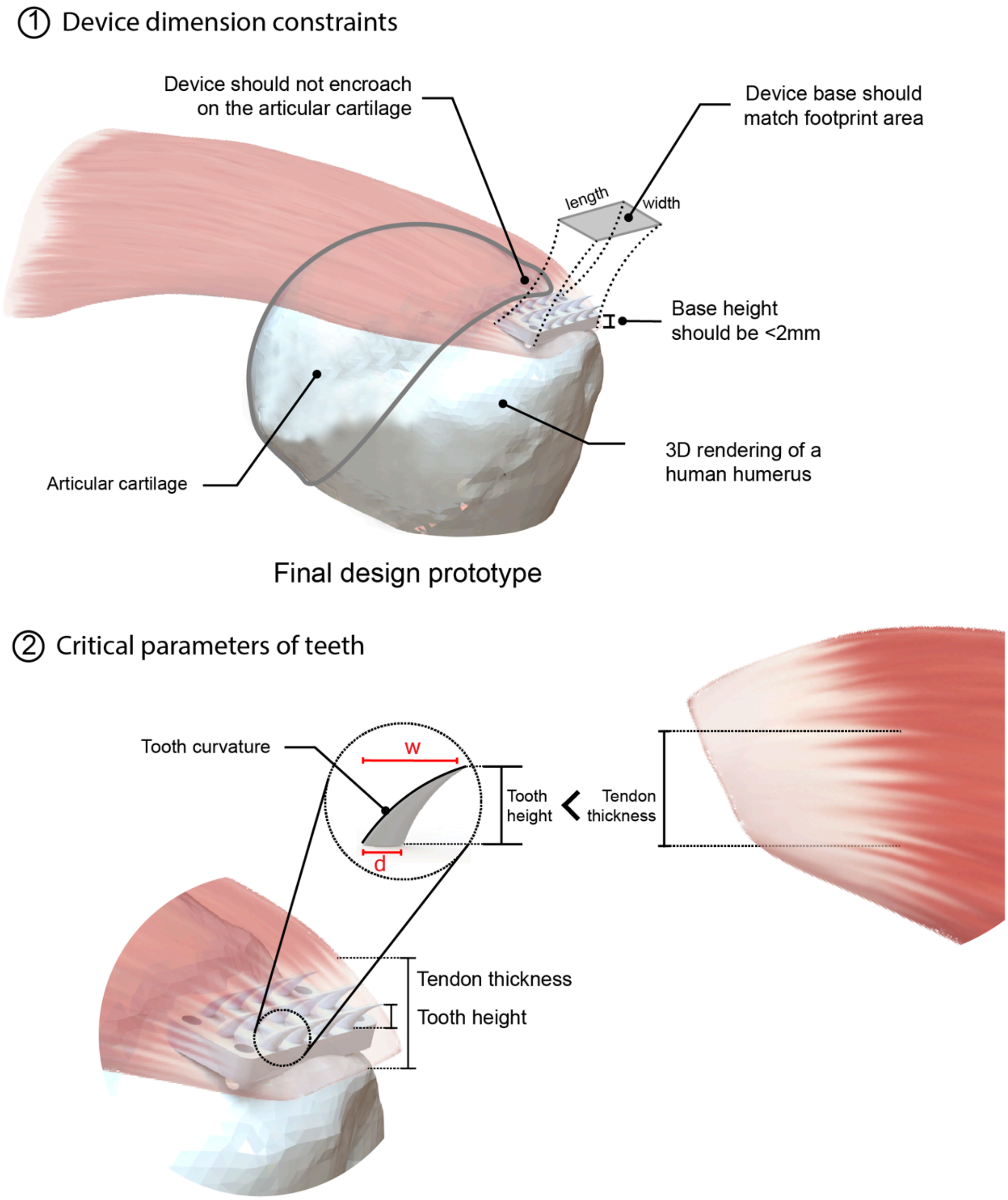

Fig. S4. Device design constraints.

The device was designed so as not to encroach on articular cartilage and should fit within the attachment footprint. The device's base was designed so as to be less than 2 mm tall, and the were designed so as to grasp the tendon without tearing through the other side.

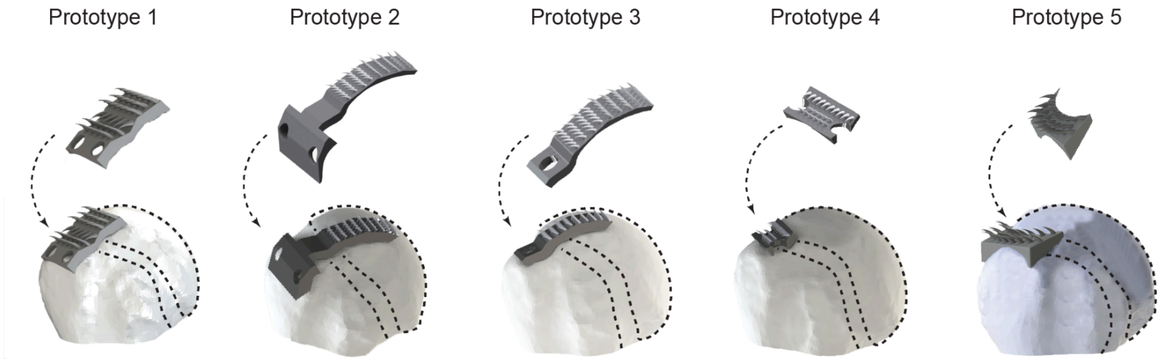

Fig. S5. Five different design iterations of the device were evaluated for fit at the attachment in biomechanics cadaver labs using shoulder cadavers

The results of these tests informed subsequent design modifications. Results confirmed that the base of the device should be constrained within the dimensions of the attachment (0.67" x 0.4" (17.0 x 10.2 mm) footprint area [*31,63*]) and not encroach on articular cartilage, and that the height of the base should be constrained to less than 2 mm.

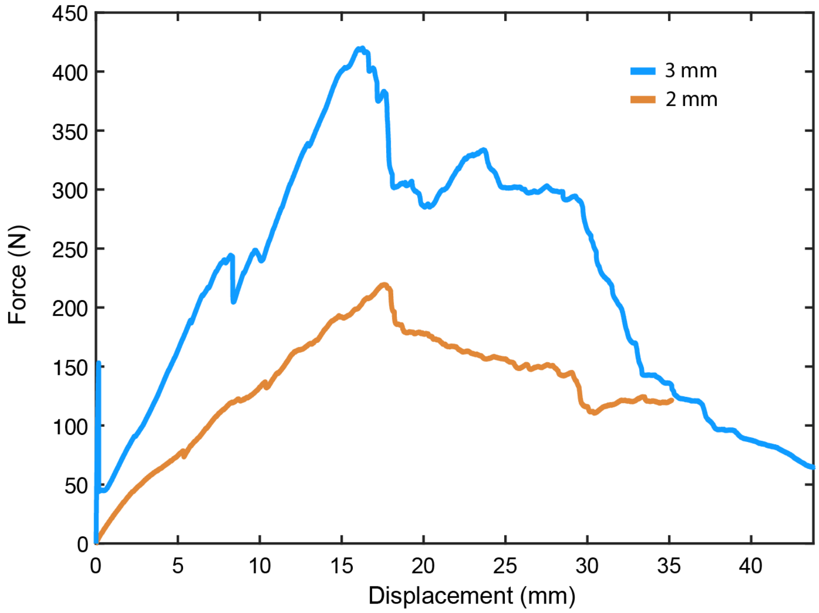

Fig. S6. Force-elongation curves of the grasping capability of two otherwise identical devices with different tooth heights (2 mm and 3 mm).

Biomechanical tests in shoulder cadavers showed that the device with larger teeth showed greater grasping capacity (i.e., higher failure load).

Fixation of the device into bone

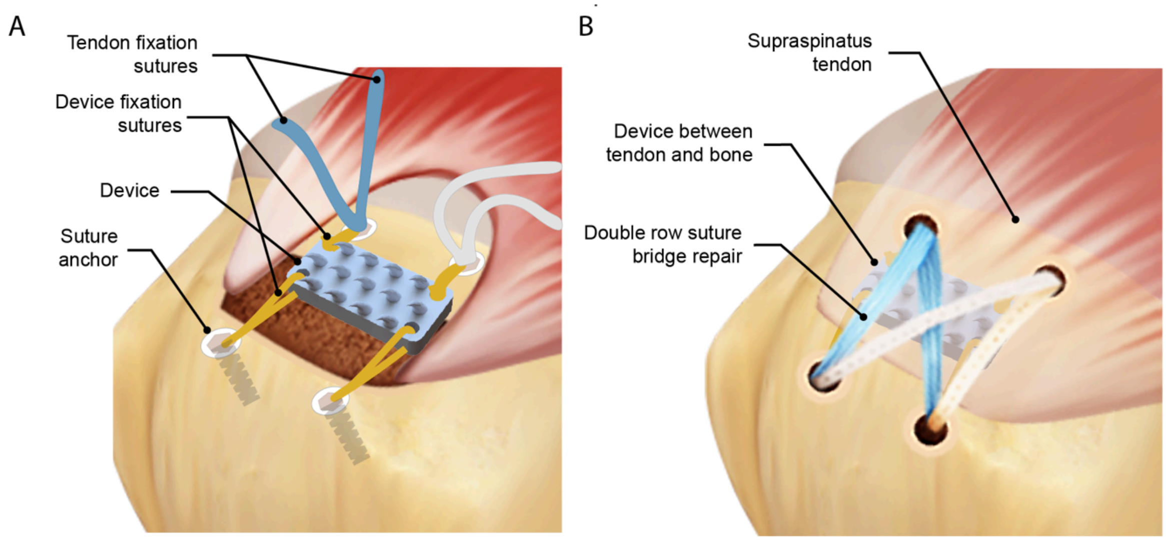

Fig. S7. Schematic of fixation of the device into bone.

(**A**) The device was secured onto bone using auxiliary sutures (yellow) connected to suture anchors. Tendon fixation sutures (blue and white) were passed through tendon and secured into bone using suture anchors in the lateral row. (**B**) The device was placed in between tendon and bone and is compatible with current double-row repair techniques.

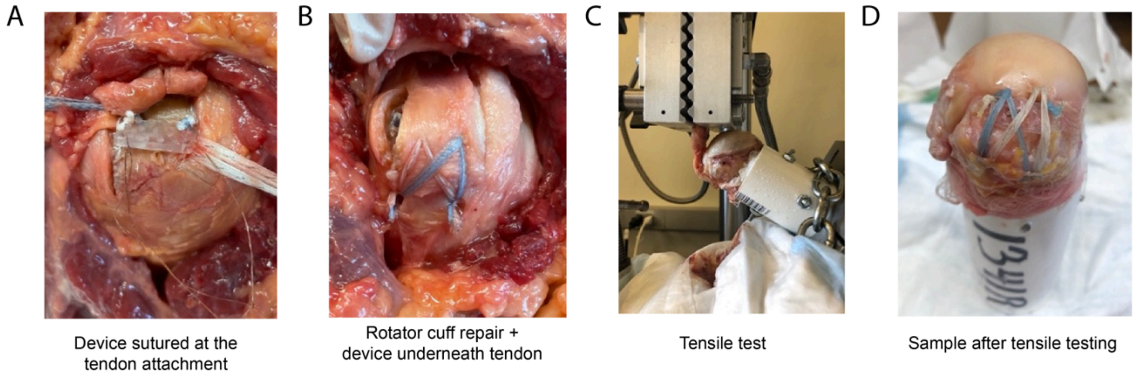

Fig. S8. Rotator cuff cadaveric repair and tensile testing setup.

(**A**). The surgeon created a massive rotator cuff tear and inserted the device into the attachment footprint. The device was fixed into the bone using additional sutures from suture anchors. (**B**) The surgeon repaired the torn tendon back to bone using the device in conjunction with the standard double-row suture-bridge technique. (**C**) Repaired supraspinatus tendons with or without the device were isolated and prepared for tensile testing. (**D**) The device after tensile testing remained intact, and no broken teeth were recorded.

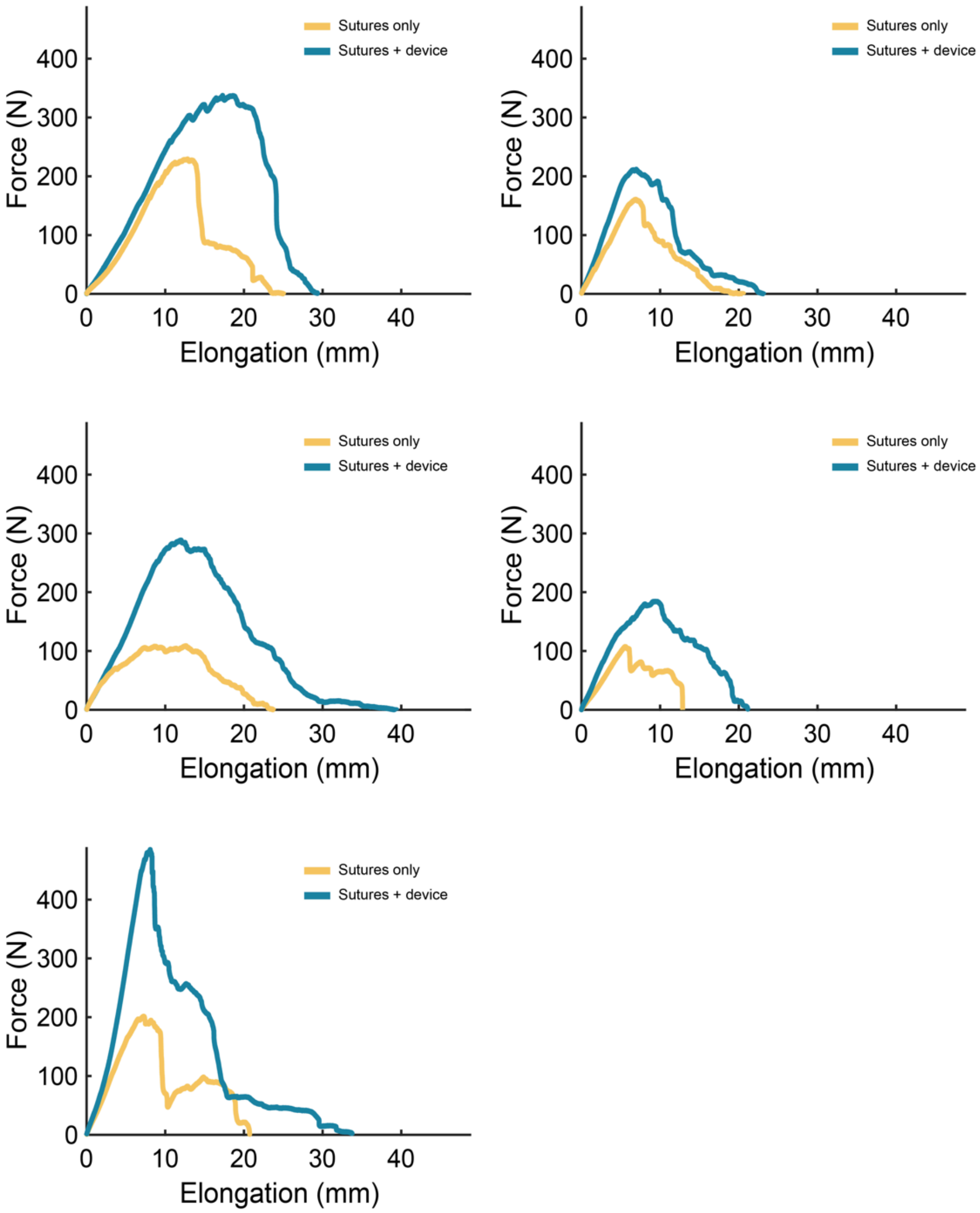

Fig. S9. Force-elongation curves of paired double-row repairs with or without the device. Each graph represents a unique pair of shoulder cadavers. Adding the device in conjunction with the double-row repair increased maximum failure load, and energy absorption (i.e., area under the curve).

Descriptions of Movies S1 – S4

Movie S1.
Representative tensile testing of single tooth gripping bovine tendon.

Movie S2.
Representative tensile testing of single tooth disengaging from bovine tendon.

Movie S3.
Representative biomechanical testing of rotator cuff repair without the device

Movie S4.
Representative biomechanical testing of rotator cuff repair with the device

Data file S1
Excel file containing data tables for all figures (adl5270_Suppl. Excel_seq1_v1.xlsx)
